# Anterior Chest Wall Reconstruction With Titanium Bars and a Rectus Abdominis Flap Following Resection of a High-Grade Sternal Neoplasm: A Case Report

**DOI:** 10.7759/cureus.117747

**Published:** 2026-10-10

**Authors:** Odrin Esfelino Ramirez Orozco, José Miguel Arriola Navas, Carlos Rolando Valladares Areano, Jennifer Rosmery Serrano Cubur, Diego Raúl Marroquín Vargas

**Affiliations:** 1 General Surgery, Hospital Roosevelt, Guatemala City, GTM; 2 Cardiothoracic Surgery, Hospital Roosevelt, Guatemala City, GTM; 3 Plastic and Reconstructive Surgery, Hospital Roosevelt, Guatemala City, GTM

**Keywords:** chest wall reconstruction, rectus abdominis flap, sternal neoplasm, sternectomy, titanium bars

## Abstract

A 75-year-old man underwent en bloc resection of a high-grade, poorly differentiated sternal neoplasm. An earlier incisional biopsy had resulted in approximately 3.5 L of blood loss, hemodynamic instability, and the need for emergency damage control surgery. The definitive procedure involved removal of the sternal body, bilateral costal cartilages, and medial insertions of both pectoralis major muscles, leaving a large full-thickness defect in the anterior chest wall. Reconstruction was undertaken in stages. After serial debridement and negative-pressure wound therapy (NPWT), skeletal stability was restored with three titanium bars and a 25 × 10 cm polypropylene mesh. A 25 × 10 cm polypropylene mesh (BioMesh; Unilene SAC, Lima, Peru), matching the dimensions of the anterior chest wall defect, was then applied over the titanium framework. The remaining pectoral tissues could not be advanced sufficiently to cover the central defect; therefore, a superiorly based right rectus abdominis muscle flap was used to provide vascularized coverage, followed by a split-thickness skin graft (STSG). Graft incorporation was estimated at 97% on postoperative day (POD) 5 and 98% on day 7. At approximately six months, the wound remained healed, the chest wall contour was preserved, and no fixation material was exposed. At 8.5 months, the chest computed tomography (CT) report described postoperative changes without a residual or recurrent mass at the operative site; the patient also reported maintaining his usual daily activities. In this setting, the pedicled rectus abdominis flap provided reliable coverage of an extensive anterior chest wall defect when conventional pectoralis advancement was not feasible and staged reconstruction was required because of the patient’s earlier physiological and wound-related complications.

## Introduction

The chest wall forms the protective framework of the thorax and consists primarily of the sternum, ribs, muscles, and overlying soft tissues. The sternum provides anterior structural support, protects the underlying heart and mediastinal organs, and contributes to respiratory mechanics. Extensive defects following sternal tumor resection therefore require reconstruction that restores chest wall stability, protects intrathoracic organs, and preserves respiratory function. Sternal resection is used for primary malignant chest wall tumors and metastatic lesions. The primary goal of surgical removal of the tumor with tumor-free margins is to achieve oncologic clearance and improve long-term outcomes; in the metastatic setting, surgery can be palliative [1]. Chest wall stability, protection of intrathoracic organs, and maintenance of respiratory mechanics are the three main goals of sternal reconstruction [2].

Materials used for chest wall stabilization are broadly classified as synthetic (alloplastic) or biological (bioprosthetic), and they differ in rigidity, protection of intrathoracic organs, preservation of muscle function, infection risk, and the extent to which they can be customized [3]. The new moldable titanium bar systems (such as the STRATOS™ system) have broadened reconstruction options. They permit rigid fixation to other anatomical structures using rib clips and plates, providing anatomical restoration and immediate stability. Titanium implants carry a risk of fracture and late displacement but offer excellent biomechanical properties for large anterior defects [4].

Another pivotal factor is soft tissue coverage. The pectoralis major flap is the first-line option because it is nearby and reliable; however, in certain scenarios, or if the tumor has invaded the muscle or prior surgery has compromised the vascular supply, this may not be possible [5,6]. In these circumstances, a rectus abdominis muscle flap, which uses vascularized muscle tissue from the abdominal wall to cover the chest wall defect, provides an alternative source of well-vascularized tissue for obliteration of dead space and durable soft tissue coverage [7]. The rectus abdominis has two dominant vascular pedicles, the superior and deep inferior epigastric vessels. Therefore, previous vessel ligation must be considered when selecting the flap configuration. An inferiorly based advancement technique has been described for selected chest wall defects [8]. We describe a reconstructive procedure for the anterior chest wall using titanium bars and a rectus abdominis flap to reconstruct an area following resection of a high-grade, poorly differentiated sternal neoplasm. This case highlights the management of an especially complex clinical case of significant biopsy-induced bleeding, polymicrobial infection, and reconstruction in a developing country.

## Case presentation

Patient information and history

A 75-year-old retired Hispanic man presented with a progressively increasing pre-sternal mass. Anemia of chronic disease with a normochromic normocytic pattern had previously been documented and treated with oral ferrous sulfate 325 mg (65 mg elemental iron) once daily for 90 days and oral folic acid 5 mg once daily for 120 days. His medical history also included chronic liver disease with features of portal hypertension and arterial hypertension for approximately 10-30 years treated with irbesartan up to 300 mg daily and type 2 diabetes mellitus for about 6-10 years treated with metformin (850 mg daily); during hospitalizations, insulin was used for glycemic control. He was classified as American Society of Anesthesiologists (ASA) physical status III, and his recorded weight was 70 kg. His performance status had not been formally assessed; however, before the complication related to the biopsy, he was able to perform all activities of daily living independently. He had no previous surgical history apart from the incisional biopsy of the sternal lesion. There was no history of previous malignancy, chemotherapy, or radiotherapy, and no known drug allergies or relevant family history was documented. Social history was limited; he denied previous tobacco and alcohol use.

Clinical findings and diagnostic assessment

The patient described a mass in the sternum that had been present for two years and was now growing rapidly. The patient had anterior chest wall pain and unquantified weight loss associated with it. He did not experience any dyspnea or respiratory restriction from the mass. Physical examination revealed an 8 x 6 cm mass with an indurated consistency, fixation to the deep planes, and trophic changes of the overlying skin at the pre-sternal site. Positron emission tomography-computed tomography (PET-CT) was unavailable because the treating institution did not have this imaging modality.

Initial biopsy and complication

The patient first underwent an incisional biopsy of the pre-sternal mass that was done via general anesthesia at the referring hospital. The procedure developed into a complication when it produced a significant amount of bleeding; the patient experienced hemodynamic instability and was estimated to have lost approximately 3500 mL of blood. Upon recognizing the severity of the complication, the patient was sent and transported to the authors’ institution for urgent evaluation and treatment.

Upon the patient’s arrival at the authors’ institution, the patient’s condition was found to include severe anemia, with his hemoglobin level being 6.9 g/dL and his hematocrit level being 20.2%, with a need for four units of packed red blood cells (PRBC). Due to the inability to achieve local control over the hemostatic process, the decision was made for emergency damage control surgery, which included sternal packing with temporary skin closure along with placing a right subclavian central venous catheter. A subsequent series of surgical procedures followed, including unpacking, applying Gelfoam® (Pfizer Inc., New York, NY), repacking, and re-closing of the chest wall. Embolization was not performed because this intervention was not available at the treating institution. Additionally, endotracheal intubation and mechanical ventilation were used for the initial stabilization of the patient. The patient was then admitted to the intensive care unit, where a thoracic CT (Figure 1) was completed while in an acute state of urgency due to the necessity to assess the nature of the lesion causing the bleeding.

**Figure 1:**
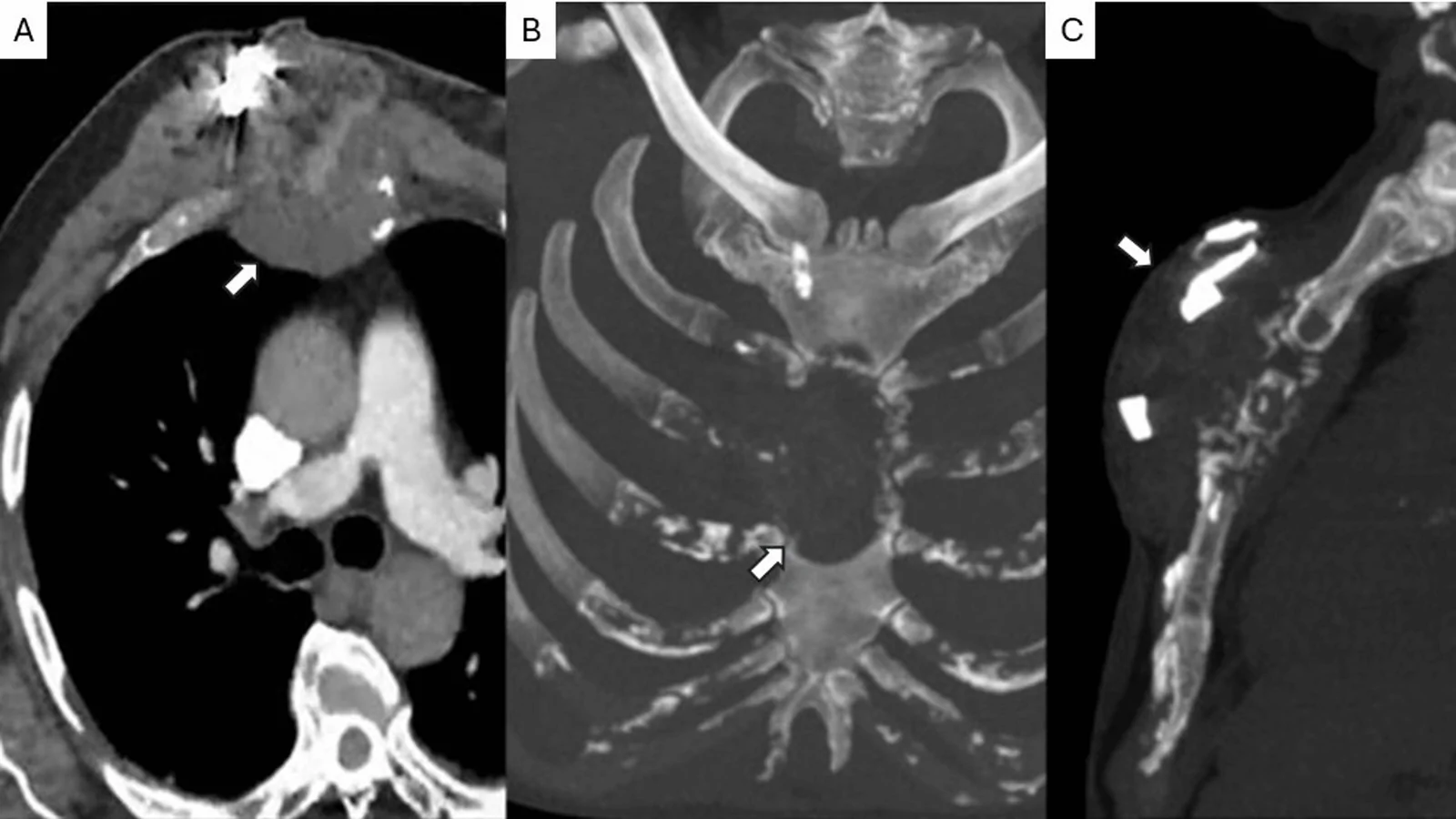
CT appearance of the destructive sternal mass during the emergency damage control period (A) Axial contrast-enhanced soft tissue window image demonstrates a large heterogeneous anterior chest wall mass centered on the sternal body, with irregular peripheral enhancement and central low-attenuation regions that may represent necrosis. The lesion extends posteriorly toward the anterior mediastinum and anteriorly into the pre-sternal subcutaneous tissues (arrow). (B) Coronal and (C) sagittal multiplanar bone window images demonstrate extensive destruction and fragmentation of the sternal body with a bulky exophytic soft tissue component (arrows). Gas locules and markedly hyperattenuating material along the superficial anterior aspect of the lesion are compatible with postprocedural changes following incisional biopsy and damage control packing. CT: computed tomography

Definitive tumor resection

Two days after damage control surgery, thoracic lavage and debridement were performed. WhiteFoam was placed, and the vacuum-assisted closure VAC® Therapy System (KCI USA, Inc., a Solventum company, San Antonio, TX) was replaced. Adequate granulation tissue was observed at the pericardial level, with closed parietal pleura and mild necrosis of the xiphoid process.

During the first post-damage control week, after clinical stabilization and control of the bleeding complication, definitive en bloc resection of the pre-sternal and sternal tumor was performed by the thoracic surgery team. The resection included the sternal body, bilateral costal cartilages, and the medial insertions of both pectoralis major muscles. Both internal thoracic arteries were identified and ligated as part of the resection. This resulted in a large full-thickness anterior chest wall defect with exposure of the pericardial surface and adjacent mediastinal tissues (Figure 2). No macroscopic invasion of the anterior mediastinal structures or suspicious regional lymphadenopathy was identified during surgery.

**Figure 2:**
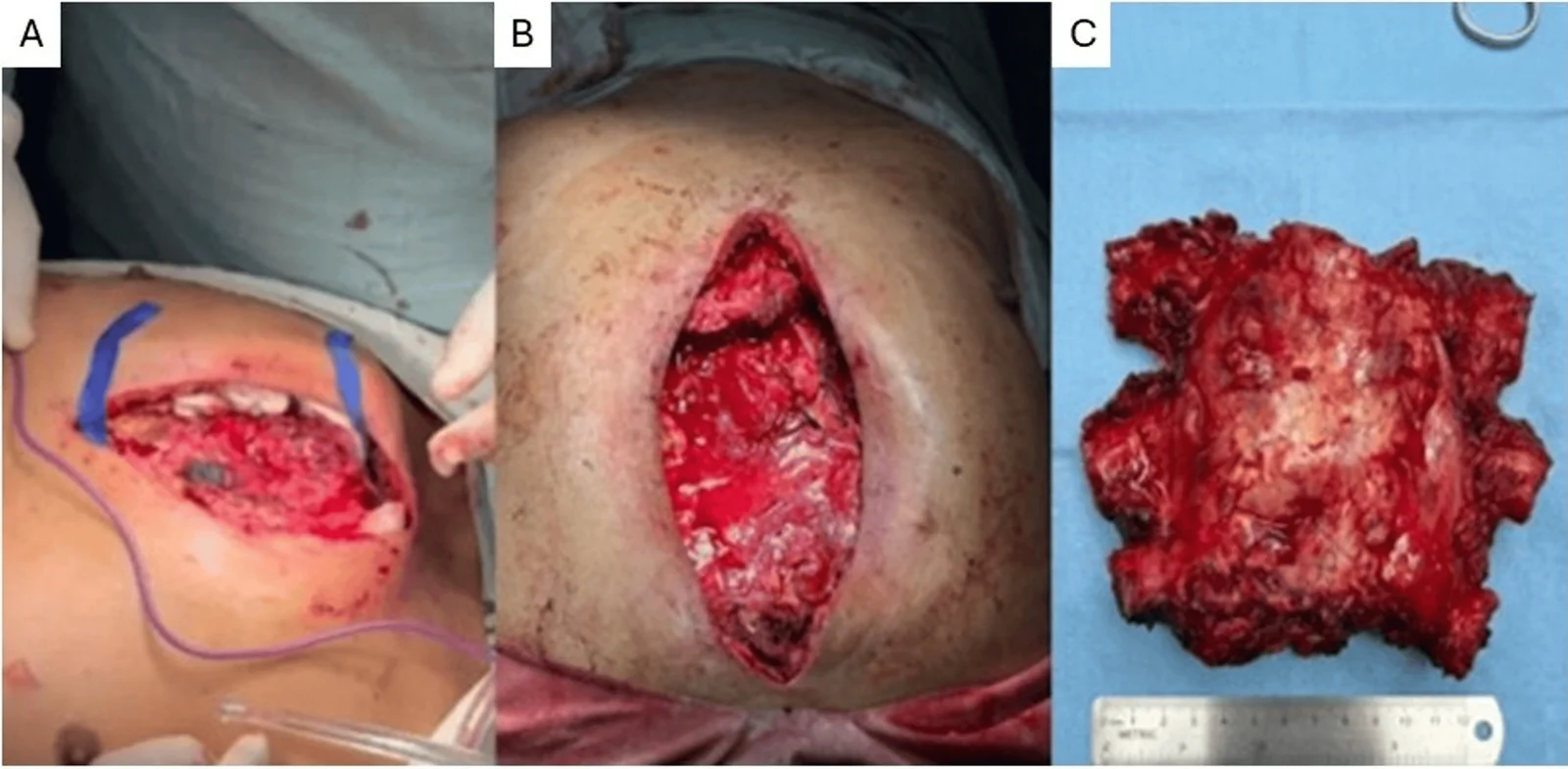
En bloc resection of the sternal neoplasm and resulting anterior chest wall defect (A and B) Intraoperative views following resection of the pre-sternal mass, sternal body, and bilateral costal cartilages demonstrating a large full-thickness anterior chest wall defect with exposure of the pericardial surface and adjacent deep mediastinal tissues. (C) Gross en bloc resection specimen containing the sternal body, attached costal cartilage segments, and surrounding soft tissue. The sternal body specimen measured 12.5 × 11.5 × 5.5 cm and contained a 4 × 3.5 cm tumor; a metric ruler is included for scale.

The surgical specimen was sent for intraoperative and definitive pathological assessment. Definitive chest wall reconstruction was deferred pending confirmation of the pathological findings and surgical margin status. The resulting defect was therefore temporarily covered with WhiteFoam and managed with negative-pressure wound therapy (NPWT). Definitive pathological examination subsequently confirmed negative surgical margins, consistent with an R0 resection.

Pathology findings

Microscopic Examination

The signed pathology diagnosis was a poorly differentiated neoplasm of the sternal body, with all evaluated proximal, distal, anterior, posterior, right lateral, and left lateral margins negative for tumor cells. The tumor exhibited a high-grade, poorly differentiated proliferation, with solid sheets and irregular nests in an infiltrative pattern. The nuclei of the neoplastic cells were pleomorphic, hyperchromatic, and irregularly chromatinized, with distinct nucleoli. There were many stained cells showing a variety of abnormal mitotic figures. Foci of necrosis, desmoplastic stromal reaction, and significant hemorrhage were observed (Figure 3).

**Figure 3:**
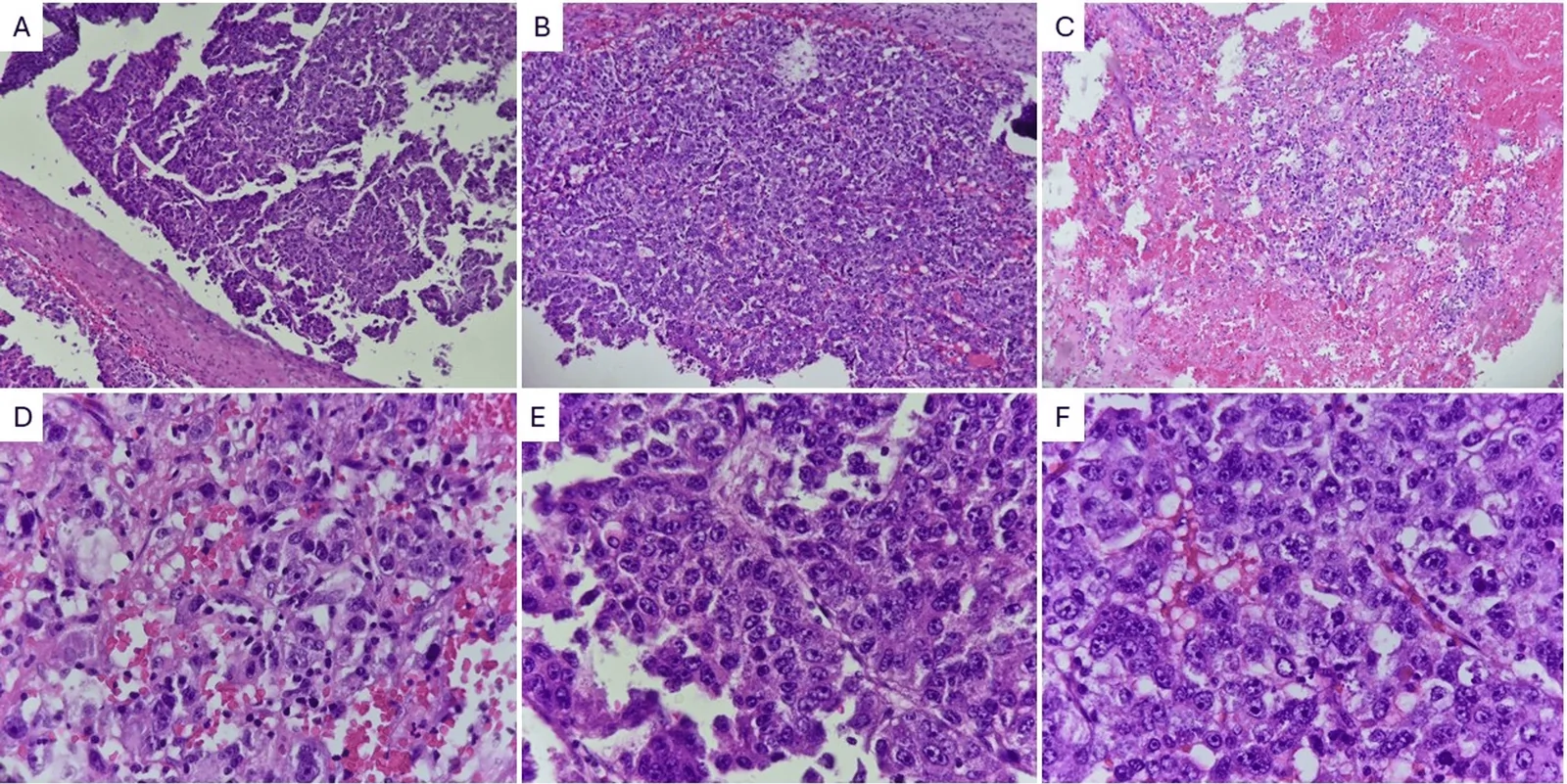
Histopathological features of the poorly differentiated neoplasm of the sternal body (H&E-stained sections) (A) Infiltrative high-grade neoplastic proliferation adjacent to and involving a trabecula of sternal bone (H&E, ×100). (B) Diffuse proliferation of neoplastic cells arranged in solid sheets and irregular nests with an infiltrative growth pattern and associated desmoplastic stromal reaction (H&E, ×100). (C) Extensive tumor necrosis and hemorrhage with residual viable neoplastic cells (H&E, ×100). (D) Pleomorphic tumor cells within a markedly hemorrhagic background (H&E, ×400). (E and F) Higher-power views show marked nuclear pleomorphism, hyperchromasia, irregular chromatin, prominent nucleoli, and scant-to-moderate pale eosinophilic cytoplasm with poorly defined cell borders (H&E, ×400). H&E: hematoxylin and eosin

Immunohistochemistry

The available immunohistochemical panel did not establish a specific line of differentiation (Table 1).

**Table 1:** Immunohistochemical profile of the sternal neoplasm Immunohistochemical staining demonstrated focal p63 positivity and a Ki-67 proliferative index of >50% of neoplastic cells. All other tested markers were negative, while PAX8 was not available for assessment. PAX8: paired box 8, CD: cluster of differentiation, SALL4: spalt-like transcription factor 4, CK5/6: cytokeratin 5/6, EMA: epithelial membrane antigen, Ki-67: Kiel-67 proliferation marker, HMB45: human melanoma black 45, SOX10: SRY-box transcription factor 10

Marker	Result
Pancytokeratin	Negative
p63	Focally positive
PAX8	Not available
CD5	Negative
CD117	Negative
Chromogranin	Negative
Synaptophysin	Negative
SALL4	Negative
p40	Negative
CK5/6	Negative
EMA	Negative
Vimentin	Negative
Ki-67	Positive in more than 50% of neoplastic cells
HMB45	Negative
Melan A	Negative
SOX10	Negative

Clinical-Oncologic Classification

The final pathological diagnosis was a high-grade, poorly differentiated neoplasm of the sternum, as the available immunohistochemical panel did not identify a specific line of differentiation. However, the lesion showed rapid clinical enlargement, extensive destruction of the sternal body on CT, and high-grade malignant features on histologic examination. Based on the primary sternal location, the aggressive radiologic appearance, the clinical progression, and the absence of a more specific diagnosis from the available immunohistochemical studies, the treating team considered osteosarcoma the most likely clinical diagnosis. However, the pathology report did not document malignant osteoid or otherwise establish osteogenic differentiation.

Serial laboratory findings

The key laboratory findings from the patient’s time in the acute phase and prior to his surgical repair are found in Table 2. The patient suffered from a significant drop in red blood cells early in the bleeding, with a hemoglobin (Hgb) of 6.9 g/dL and some decrease in platelets at that time, being slightly low at 132 × 10³/uL. Approximately two weeks before flap coverage, after rigid chest wall stabilization, there were several abnormalities noted on laboratory work. He had a high white blood cell count along with neutrophilic predominance. His blood sugar was significantly elevated at 325 mg/dL. His sodium was mildly decreased at 132 mmol/L. Liver enzyme levels, specifically aspartate aminotransferase (AST), alanine aminotransferase (ALT), and gamma-glutamyl transferase (GGT), were elevated. By one week prior to definitive flap coverage, abnormal laboratory results were back to normal or near normal. However, he was still anemic, but it was improving; his Hgb had risen to 10 g/dL. At no point did the patient’s renal function decline in relation to his blood urea nitrogen (BUN) and serum creatinine.

**Table 2:** Laboratory findings during the emergency period and before definitive chest wall reconstruction NR: not reported, GFR: glomerular filtration rate, AST: aspartate aminotransferase, ALT: alanine aminotransferase, GGT: gamma-glutamyl transferase, LDH: lactate dehydrogenase, INR: international normalized ratio, WBC: white blood cell count, mg/dL: milligrams per deciliter, g/dL: grams per deciliter, mmol/L: millimoles per liter, U/L: units per liter, mL/min/1.73 m²: milliliters per minute per 1.73 square meters, µL: microliter ↑ indicates a value above the reference interval, and ↓ indicates a value below the reference interval.

Laboratory parameter	Reference interval	Emergency period	15 days before reconstruction	14 days before reconstruction	13 days before reconstruction	7 days before reconstruction
Hematology						
White blood cell count, ×10³/µL	3.4-9.6	NR	NR	11.65 ↑	7.0	5.7
Absolute neutrophil count, ×10³/µL	1.56-6.45	NR	NR	9.81 ↑	NR	NR
Absolute lymphocyte count, ×10³/µL	0.95-3.07	NR	NR	1.03	NR	NR
Hemoglobin, g/dL	13.2-16.6	6.9 ↓	NR	NR	9.4 ↓	10.0 ↓
Hematocrit, %	38.3-48.6	20.2 ↓	30.7 ↓	NR	28.0 ↓	30.8 ↓
Platelet count, ×10³/µL	135-317	132 ↓	246	NR	183	264
Renal and metabolic profile						
Glucose, mg/dL	70-140	NR	NR	325 ↑	119	108
Creatinine, mg/dL	0.74-1.35	1.29	NR	1.17	0.78	0.70 ↓
Blood urea nitrogen, mg/dL	8-24	20	NR	25.2 ↑	21.5	13
Estimated GFR, mL/min/1.73 m²	≥60	NR	NR	62	NR	NR
Sodium, mmol/L	135-145	139	NR	132 ↓	133 ↓	136
Potassium, mmol/L	3.6-5.2	4.2	NR	3.7	4.4	4.1
Liver biochemistry						
Total bilirubin, mg/dL	0.0-1.2	NR	NR	0.60	NR	NR
Direct bilirubin, mg/dL	0.0-0.3	NR	NR	0.46 ↑	NR	NR
AST, U/L	8-48	NR	NR	88.3 ↑	NR	NR
ALT, U/L	7-55	NR	NR	72.2 ↑	NR	NR
GGT, U/L	8-61	NR	NR	360 ↑	NR	NR
LDH, U/L	122-222	NR	NR	183	NR	NR
Coagulation						
Prothrombin time, seconds	9.4-12.5	NR	NR	11.9	11.2	12.0
INR	0.9-1.1	1.19 ↑	NR	1.08	1.02	1.09
Activated partial thromboplastin time, seconds	25-37	NR	NR	33.7	35.3	32.9
Fibrinogen, mg/dL	200-393	NR	NR	310	NR	NR

Post-resection management and staged chest wall reconstruction

The initial approach to address the anterior thoracic defect involved repeated washouts, multiple surgeries/debridement procedures for both mechanical and surgical removal of necrotic debris, application of WhiteFoam dressing, and application of negative-pressure wound therapy (NPWT) via VAC® Therapy System (KCI USA, Inc., a Solventum company, San Antonio, TX). Each time the surgeon examined tissue viability in addition to granulation at the pericardial surface and wound margin, the amount of remaining contamination, as well as the size of the mild xiphoid necrosis, was noted.

*Proteus mirabilis* and New Delhi metallo-β-lactamase-producing *Klebsiella pneumoniae* were cultured approximately 3.5 weeks prior to reconstruction day 0, when tissue samples were taken for evaluation and culture during wound management. The principal antibiotic regimen included 14 days of amikacin 750 mg iv q24h, meropenem 1 g iv q8h, and polymyxin B 750,000 units iv q12h with an initial dose and then every 12 hours. In the post-discharge phase, ampicillin/sulbactam was administered during the outpatient follow-up of the wound care process.

Rigid chest wall reconstruction

Approximately three weeks prior to achieving final soft tissue closure, the anterior chest wall was reconstructed by the thoracic surgery team using three titanium rods from the STRATOS system (MedXpert GmbH, Heitersheim, Germany). The two rods used were placed in a crossed manner, and the third rod was placed horizontally, creating an asterisk pattern. This arrangement was selected because, in the surgical team’s intraoperative assessment, it provided the best rigid support for the anterior chest wall. The polypropylene mesh used was BioMesh (Unilene SAC, Lima, Peru), measuring 25 × 10 cm, corresponding to the dimensions of the anterior chest wall defect. It was applied on top of the titanium framework. The treating thoracic surgeon had previous experience placing mesh anterior to titanium bars following rib resections for osteochondromas. This experience informed the use of the same configuration for the anterior chest wall reconstruction in this patient. Anterior placement was selected with the intention of promoting granulation tissue formation over the mesh during negative-pressure wound therapy and facilitating its integration with the superior, inferior, and lateral margins of the defect. A posterior mesh layer was not added because the chosen approach focused on this anterior tissue integration. Following the application of the mesh, negative-pressure wound therapy (NPWT) was applied again as the wound bed was prepared for vascularized coverage. The staged bar reconstruction process, mesh placement, and initial application of the temporary NPWT dressing are illustrated in Figure 4.

**Figure 4:**
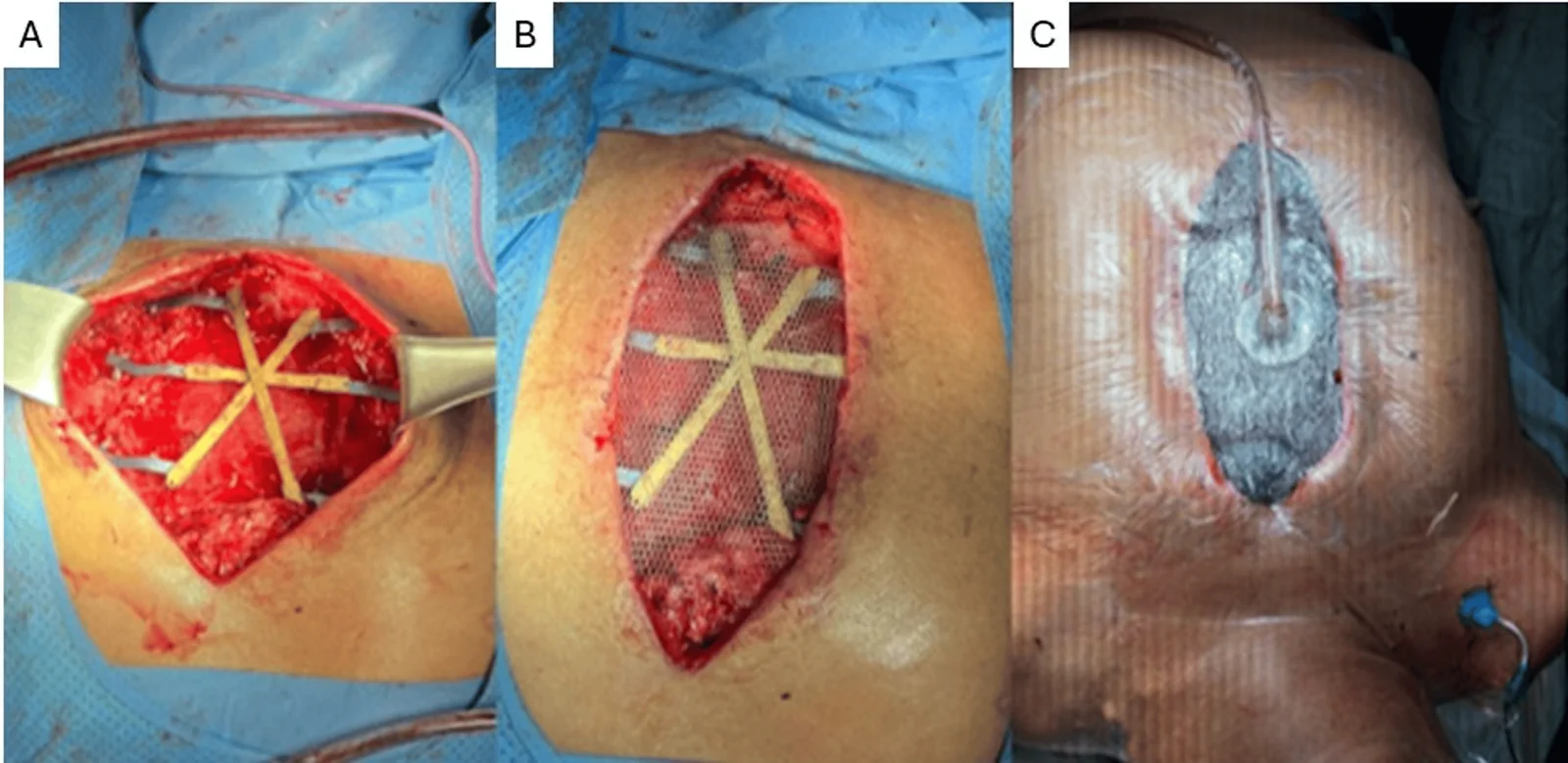
Staged rigid reconstruction of the anterior chest wall (A) Three titanium bars were fixed to the residual costal arches: two in a crossed orientation and one transversely forming an asterisk-like configuration to provide structural stabilization. (B) A 25 × 10 cm polypropylene mesh was positioned over the titanium construct to reinforce the reconstruction. (C) The reconstructed wound was temporarily covered with a negative-pressure wound therapy dressing pending definitive vascularized soft tissue coverage.

Pre-reconstructive wound bed preparation and clinical optimization

At the time of the second evaluation, about two and one-half weeks prior to the reconstruction day 0, the titanium fixation bar and polypropylene mesh (mesh) appeared to remain intact and in acceptable condition; however, granulating tissue was beginning to form along both the cephalad (top) and lateral (side) wound edges. During that week, laboratory tests indicated leukocytosis with neutrophil predominance, significant elevation in blood sugar, slight decrease in sodium levels, persistent anemia, and increases in aspartate aminotransferase, alanine aminotransferase, direct bilirubin, and gamma-glutamyl transferase. The renal functions were maintained; however, there were no substantial prolongations in the available coagulation studies.

The main focus of care during this period included management of possible infection, optimization of glycemic and metabolic states, and further preparation of the wound bed for future reconstructive efforts. Approximately one week prior to the reconstruction day 0, the fixation bars had been left exposed, but they had adequate amounts of granulation tissue forming around them. A mechanical debridement of the wound margins and a new application of VAC dressings occurred. At this point in time, the white cell count, serum glucose, and sodium values had returned to normal ranges; however, the patient still had persistent anemia.

Definitive vascularized soft tissue reconstruction

On reconstruction day 0, the precordial defect had an approximate size of 25 x 10 cm. Although the titanium fixative material could still be seen on the surface of the wound bed, there was enough granulation present to allow for a successful reconstructive surgery. Because the medial insertions of both pectoralis major muscles had been resected during tumor resection, the remaining pectoral tissue was not sufficient for conventional advancement over the defect. A superiorly based right rectus abdominis muscle flap was therefore selected for coverage by the plastic surgery team, characterized as a type III flap according to the Mathes and Nahai classification [5], which was then elevated as a superiorly based pedicled muscle flap. The inferior epigastric vessels were identified and ligated, and the muscle was divided caudally. The flap was mobilized cephalad, positioned over the titanium bar and polypropylene mesh construct, and secured to the remaining pectoral tissues.

Next, a split-thickness skin graft (STSG) was harvested from the patient’s left thigh and draped over the muscle that had been transplanted. The donor site was covered with a hydrocolloid wound dressing, which was removed on postoperative day (POD) 3 without documented donor site complications. The STSG was sutured into place using both staples and sutures. Petroleum gauze was applied over the graft, and negative-pressure wound therapy (NPWT) was initiated. Two different types of suction drainage systems were also used, including Hemovac and Jackson-Pratt drains. Finally, a synthetic prosthetic material was applied to reinforce the donor site where the abdominal fascia had been removed. No intraoperative complications were documented. Flap harvest and cephalad mobilization are shown in Figure 5. Figure 6 shows the transfer and inset of the flap and placement of the split-thickness skin graft.

**Figure 5:**
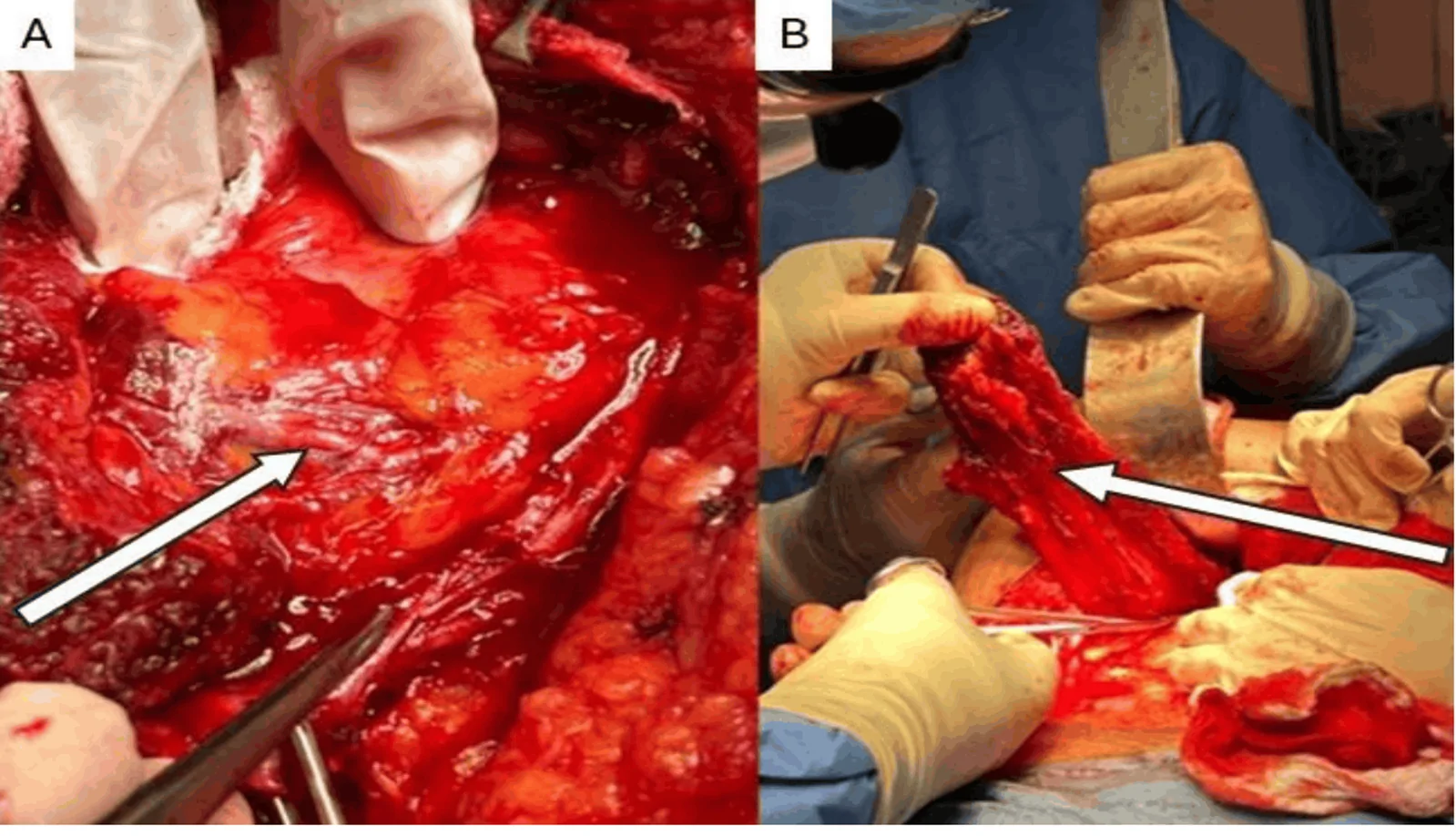
Harvest and cephalad mobilization of the right rectus abdominis muscle flap (A) Intraoperative identification of the inferior epigastric vessels before ligation (arrow). (B) Following ligation of the inferior epigastric vessels and caudal division of the rectus abdominis muscle, the flap was mobilized cephalad toward the anterior chest wall defect (arrow), retaining its superior attachment.

**Figure 6:**
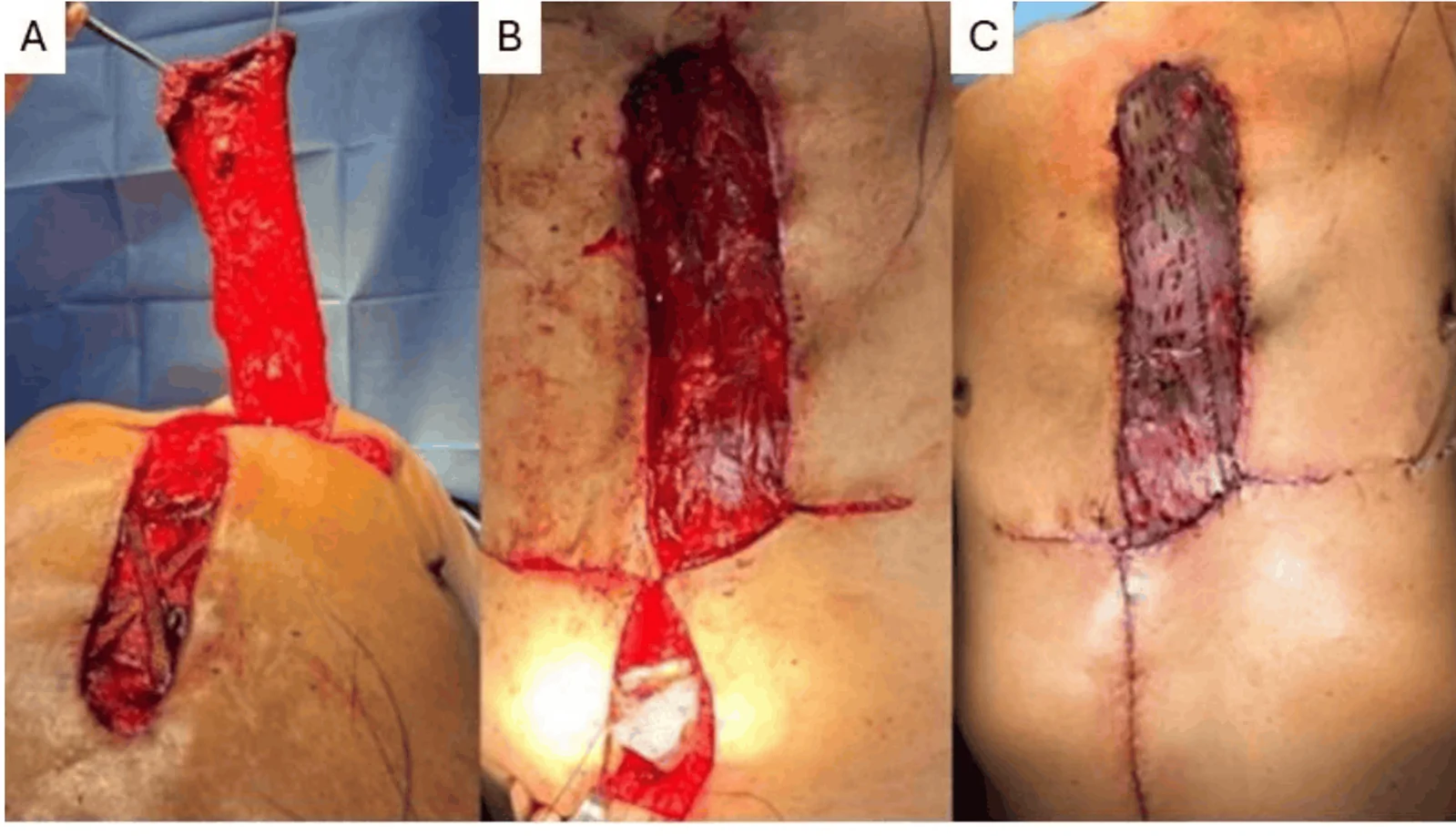
Definitive vascularized soft tissue reconstruction of the anterior chest wall (A) Elevation of the superiorly based right rectus abdominis muscle flap with preservation of its superior vascular pedicle. (B) The flap was mobilized cephalad and inset over the anterior chest wall defect, providing vascularized coverage of the titanium bar and polypropylene mesh construct. (C) A split-thickness skin graft harvested from the left thigh was secured over the muscle flap to complete the reconstruction.

Postoperative systemic therapy

Systemic treatment started in the hospital on postoperative day 5 with paclitaxel at a dose of 110 mg and carboplatin at a dose of 200 mg. Early initiation was chosen because of the rapidly progressive nature of the tumor and the treating oncology team’s recommendation. Treatment began only after the skin graft showed approximately 97% take on postoperative day 5, with no secretion, hematoma, or clinical signs of infection, and blood counts considered adequate for chemotherapy.

The treatment record documents one full cycle as three identical doses (same) given on treatment day 1, day 8, and day 15. There were no immediate complications noted to have occurred after any of these treatments. No additional chemotherapy cycles could be confirmed. Radiotherapy was recommended as part of the oncologic treatment plan but was not administered because it was unavailable at the treating institution; the patient therefore received chemotherapy alone.

Postoperative course and follow-up outcomes

Early Postoperative Reconstructive Course

Beginning on postoperative day (POD) 5, the negative-pressure wound therapy (NPWT) dressing was removed. Approximately 97% of the skin graft had been incorporated into the underlying tissue; there was no secretion, hematoma, clinical infection, or evidence that wound margins had become compromised. On POD 6, the patient received a single compatible O-positive packed red blood cell unit. No transfusion reaction was documented. At POD 7, the percentage of skin graft incorporation into the underlying tissue increased to approximately 98%. The wound was still clean with no secretion present, and the sutures were intact. The patient was discharged from the surgical service on POD 12.

Subsequent Outpatient Wound Healing

At each outpatient follow-up visit, there was a continued progression toward complete healing of the skin graft. The pedicle flap appeared to be incorporated into the remaining tissue very well; the appearance of the scars was good (no swelling or redness), and clinically, it appeared to have no infection. One small (less than 0.5 cm) area on the top of the pedicle flap that had been left open as part of the original surgical procedure remained slightly semi-dehisced; this site continued to produce minimal amounts of serous fluid and has since been treated with standard local wound management techniques using silver nitrate and calcium alginate dressings. There was also no evidence of any exposed suture materials at the time of the last reconstructive assessment.

Medium-Term Radiologic, Reconstructive, and Functional Outcomes

Approximately 4.5 months after reconstruction day 0, contrast-enhanced thoracic CT revealed that the osteosynthesis material involved the second, third, and fourth costal arches and the sternum. The stabilization bar for this retained three-bar construct crossed over both the manubrium and the sternal area. There was no radiologic indication of loosening (Figure 7A). The patient’s rectus abdominis muscle flap and overlying skin graft were clinically stable at approximately six months post-op. The anterior chest wall contour appeared to have been maintained; no hardware was visible externally, and there was no clinical evidence of an active infection at the site of the wound closure (Figure 7B).

**Figure 7:**
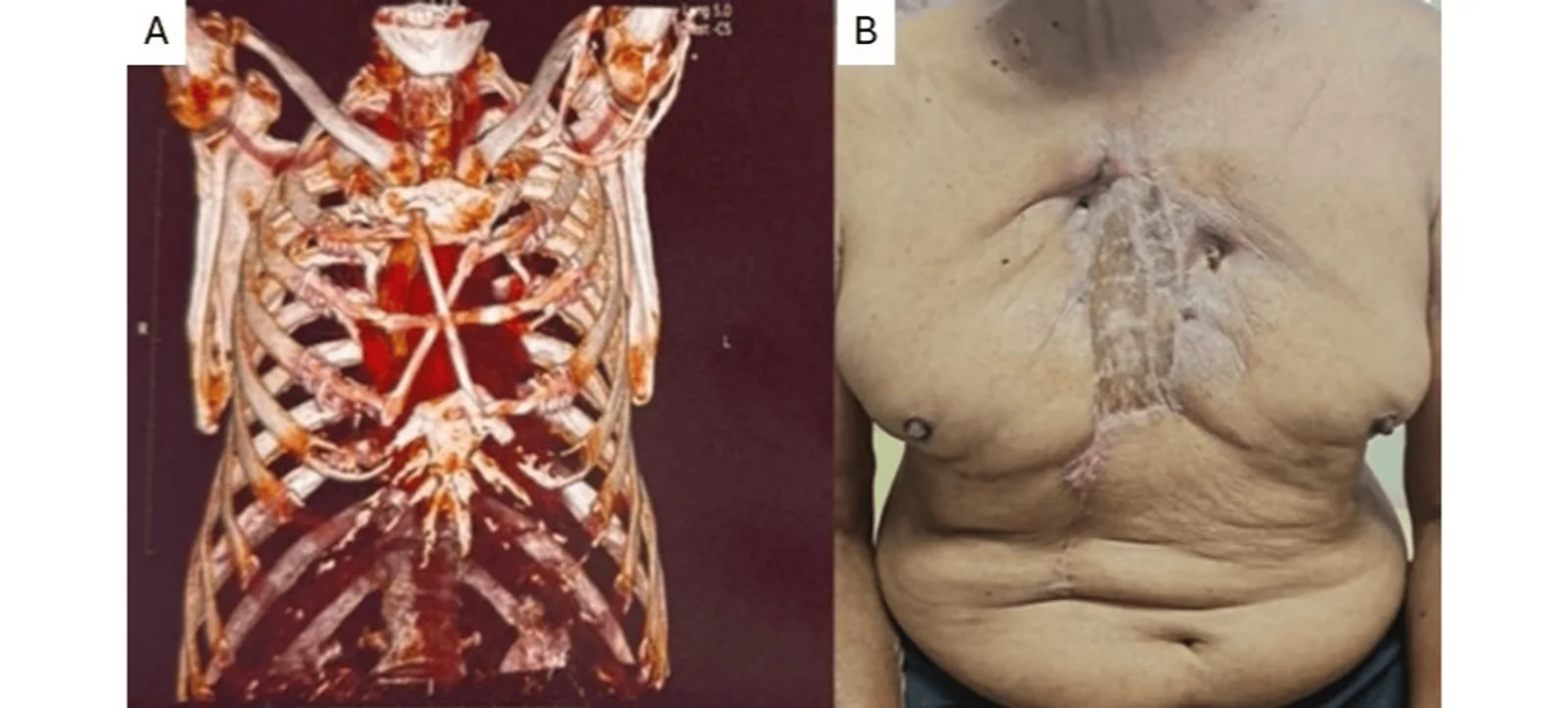
Medium-term structural and soft tissue outcomes after anterior chest wall reconstruction (A) Three-dimensional volume-rendered CT obtained about 4.5 months after reconstruction shows the retained three-bar titanium construct involving the second through fourth costal arches and sternum, with no radiologic evidence of loosening. (B) A clinical photograph at approximately six months shows a healed rectus abdominis flap with split-thickness skin graft coverage, preserved anterior chest wall contour, and no exposed hardware or clinical evidence of local infection. CT: computed tomography

Follow-Up at 8.5 Months

At 8.5 months after final reconstructive surgery, a CT scan of the chest using non-contrast imaging revealed that the proximal and middle portions of the sternal body were absent, with preservation of the distal body and manubrium. There was also bilateral osteosynthesis material connecting the second through fourth ribs. The radiologist’s report stated that there were no residual tumors or recurrent tumor(s) in the operative area, pulmonary nodules or masses, or hilar or mediastinal lymph node involvement. The left base and right middle lobe had evidence of pleuropulmonary scarring due to previous disease, but there was no pleural fluid present (Figure 8).

**Figure 8:**
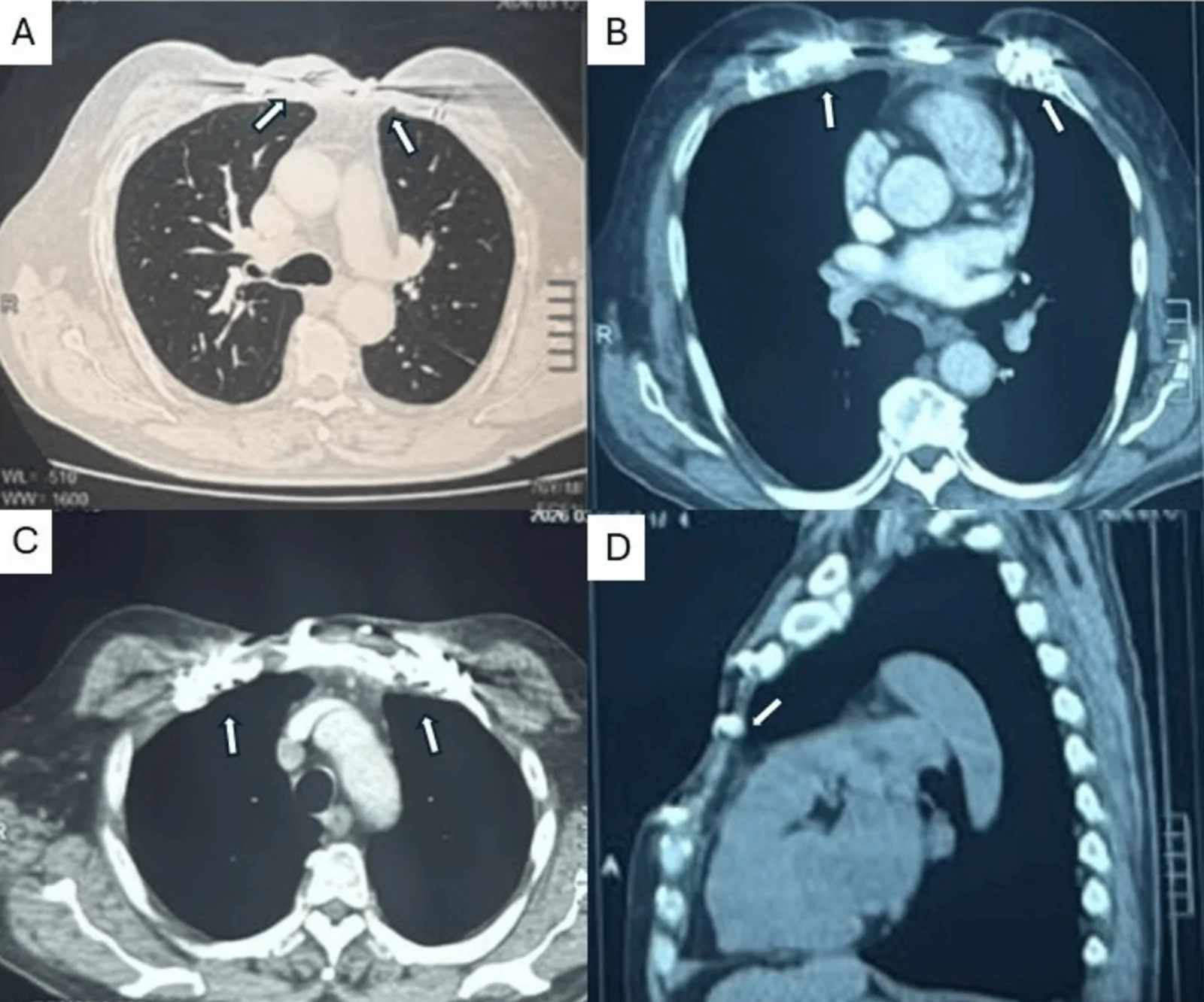
Follow-up thoracic CT 8.5 months after anterior chest wall reconstruction (A) Axial lung window image at the carinal level showing aerated lung parenchyma and postoperative anterior chest wall changes (arrows). (B and C) Axial contrast-enhanced soft tissue window images at cardiac and upper mediastinal levels, respectively, demonstrating anterior metallic fixation components, associated streak artifact, and overlying soft tissue (arrows). (D) Sagittal CT image showing the anterior chest wall contour and its relationship to the underlying heart and mediastinum (arrow). CT: computed tomography

By the time of follow-up, the patient stated that he was capable of carrying out all his normal day-to-day functions independently without experiencing undue respiratory limitation from performing these. Additionally, the patient reported being able to cycle comfortably. Therefore, based on this information, the patient had good functional tolerance to moderate levels of physical activity. However, no assessment of lung volume or diffusion capacity for carbon monoxide by spirometry, nor any other assessments of chest wall mechanics or cardiopulmonary reserve through cardiopulmonary exercise testing or standard measures of chest wall mechanics, was completed. The CT report at 8.5 months described no residual or recurrent mass at the operative site. The clinical course from initial presentation through the latest available follow-up is summarized in Table 3.

**Table 3:** Key clinical timeline from symptom onset through the last available follow-up CT: computed tomography, ICU: intensive care unit, NDM: New Delhi metallo-β-lactamase, NPWT: negative-pressure wound therapy, RBC: red blood cells

Relative period	Clinical event
Approximately 2 years before presentation	A pre-sternal mass developed and subsequently enlarged rapidly
At presentation	Painful, indurated approximately 8 × 6 cm anterior chest wall mass
Initial biopsy	Major bleeding; approximately 3,500 mL blood loss; 4 units packed RBCs
Emergency period	Damage control surgery, intubation, mechanical ventilation, ICU transfer
2 days later	Thoracic lavage, debridement, WhiteFoam, and VAC therapy
First post-damage control week	En bloc sternal and chest wall tumor resection
Subsequent wound management period	Serial debridement and negative-pressure therapy
Approximately 3.5 weeks before definitive coverage	*Proteus *mirabilis and NDM-producing K. pneumoniae documented
Approximately 3 weeks before definitive coverage	Three titanium bars and 25 × 10 cm polypropylene mesh placed
Approximately 2 weeks before definitive coverage	Continued antimicrobial treatment and metabolic optimization
Reconstruction day 0	Right rectus abdominis muscle flap and left thigh split-thickness skin graft
Postoperative day 5	Approximately 97% graft integration; paclitaxel 110 mg + carboplatin 200 mg
Postoperative day 6	1 unit of O-positive packed RBC transfused
Postoperative day 7	Approximately 98% graft integration
Postoperative day 12	Discharge
Approximately 4.5 months after reconstruction	CT showed maintained fixation without loosening and no pulmonary nodules/masses
6 months after reconstruction	Clinical assessment and photograph showed healed flap and skin graft coverage without exposed hardware
8.5 months after reconstruction	The latest available thoracic CT reported no residual or recurrent mass at the operative site

## Discussion

Extensive sternal resection creates a composite defect in which oncologic clearance must be balanced with protection of the mediastinal structures, restoration of chest wall stability, and provision of durable vascularized coverage. In this patient, life-threatening biopsy-related hemorrhage, a culture-positive wound, and unresolved histologic lineage compounded these priorities. This flap represented a suitable reconstructive option, as resection of the medial insertions of both pectoralis major muscles made it impossible to perform a traditional advancement flap using the pectoralis muscles over the middle area of the defect. In addition, both internal thoracic arteries had been ligated during the en bloc resection, further altering the local vascular anatomy and limiting the internal thoracic vessels’ availability as conventional recipient vessels for free tissue transfer. Furthermore, due to the extensive earlier bleeding that had caused hemodynamic instability along with the requirement for a damage control surgical approach, a stable regional option was desired to minimize operative complexity. This case is clinically instructive because current consensus and reconstructive reviews support individualized reconstruction for extensive anterior defects while acknowledging that no material or flap has demonstrated universal superiority [9,10].

Diagnostic and oncologic interpretation

Histopathological evaluation revealed an aggressive, poorly differentiated malignancy with significant nuclear variability (pleomorphism), increased nuclear density (hyperchromasia), and abnormal mitotic activity. The presence of focal necrosis and hemorrhage along with a dense desmoplastic stromal reaction further supported this conclusion. Proliferation potential was also confirmed to be high, as evidenced by >50% of the neoplastic cell population staining for Ki-67; however, no evidence of lineage could be established based on the immunohistochemical results. Along with the Ki-67 staining, p63 was also found to be focally positive. However, all of the antibodies that were tested for epithelial, neuroendocrine, germ cell, and melanocytic lineages were either negative or not available. Due to its location within the sternum, radiographic findings indicative of bone destruction, rapid growth, and highly aggressive histology, the treatment team believed that osteosarcoma was the most likely clinical working diagnosis. However, no evidence of malignant osteoid formation or confirmation of osteogenic lineage was documented in the pathology report.

Strauss et al. [11] recommend that suspected primary bone sarcomas be evaluated within a specialist sarcoma network and reviewed by a pathologist experienced in bone tumors. Ancillary immunohistochemical and molecular studies may assist classification when conventional findings are inconclusive. In the present case, the available evidence confirms a high-grade malignant neoplasm but does not permit confident assignment to a specific histologic subtype. All resected margins were free of cancerous growth. Wang et al. [9] suggest that it is possible to attempt achieving a surgical margin larger than 2 cm on chest wall tumors as long as vital structures are preserved. Focal necrosis and hemorrhage, along with a dense desmoplastic stromal reaction, were also present. Approximately 3,500 mL of estimated bleeding caused unstable hemodynamics, required transfusions of four units of PRBCs, and required damage control surgery, mechanical ventilation, and intensive care. Although considerable hemorrhage was found throughout the resection specimen, an angiographic evaluation or conclusive evidence of either tumor hypervascularity or vascular invasion by the tumor could not be obtained. Guidance for managing bone sarcomas is to coordinate biopsy with other aspects of the team’s care plan. In the majority of cases, image-guided core needle biopsies can be performed as long as there is enough room after the biopsy for surgical resection [11]. While these guidelines do not prove that another type of procedure would have stopped the bleeding from happening in this case, they emphasize the importance of multidisciplinary planning to manage the complexities associated with anatomical tumors at the sternum.

Rigid reconstruction and implant-related considerations

The approximately 25 × 10 cm anterior defect, bilateral costal cartilage resection, and exposure of pericardial and mediastinal surfaces provided a reasonable anatomical indication for rigid stabilization. Wang et al. [9] recommend rigid reconstruction when the maximum diameter of an adult chest wall defect exceeds 5 cm. The authors acknowledge, however, that this recommendation is based predominantly on expert consensus because high-quality comparative evidence remains limited. Selection between rigid and semirigid reconstruction remains individualized according to defect size and location, extent of resection, residual tissue quality, proximity to intrathoracic structures, material availability, and institutional experience. Aranda et al. [10] describe this distinction as an unresolved issue and suggest reserving rigid constructs primarily for extensive defects. Reported advantages of titanium include biocompatibility, osseointegration, relative resistance to infection, a high strength-to-weight ratio, and low radiographic density. Nevertheless, rupture, displacement, infection, erosion, and chronic pain remain recognized complications. Three titanium bars were secured to the residual costal arches, with two crossing diagonally and a third positioned transversely. The resulting asterisk-like configuration was judged intraoperatively by the surgical team to provide the best rigid support for this patient’s anterior chest wall defect. A 25 × 10 cm polypropylene mesh was then placed over the construct.

Clermidy et al. [12] evaluated 68 patients who underwent malignant chest wall resection and reconstruction with titanium bars or sternal plates. During a mean follow-up of 43.2 months, no patient developed paradoxical chest wall motion. Surgical site infection occurred in 12 (18%) patients, and six of these infections required removal of fixation material. Overall, 7 (10%) patients underwent bar or plate removal, implant failure occurred in 4 (6%), and chronic pain requiring analgesic treatment for at least three months was reported in 16 (24%) [12]. These results indicate that titanium implants are capable of providing structural stability; however, there is a necessity for continued longitudinal monitoring. The lack of loosening observed on CT scans at approximately 4.5 months post-reconstruction suggests initial stabilization of fixation but does not predict potential long-term issues such as chronic pain or late-onset implant-associated infection.

Wound management and infection control

Cultures obtained during wound management grew *Proteus mirabilis* and New Delhi metallo-β-lactamase-producing *Klebsiella pneumoniae*. The principal antimicrobial course lasted 14 days and included amikacin, meropenem, and polymyxin B; ampicillin/sulbactam was prescribed later during outpatient wound care. The anatomical extent of infection remained uncertain. Thoracic computed tomography raised concern for anterior mediastinitis, but the records did not establish that cultures were obtained directly from mediastinal tissue or fluid. Infection involving the titanium bars or polypropylene mesh was likewise not confirmed. The findings are therefore most accurately described as a culture-positive wound with radiologic concern for mediastinal involvement.

Lampridis et al. [13] reported that local infection occurs in approximately 2%-23% of patients after chest wall resection and reconstruction, with the variation reflecting differences in patient populations and definitions of infection. Management may require antimicrobial therapy, drainage, repeated debridement, elimination of dead space, NPWT, and vascularized tissue coverage. The same review summarized a retrospective cohort of 86 patients who underwent chest wall tumor resection and reconstruction. Seven developed local sepsis requiring reoperation. Vacuum-assisted therapy contributed to complete wound healing in all seven over a median period of 14 months, although prosthetic material required removal in four [13]. These observations suggest that NPWT can support infection control and wound bed preparation but does not guarantee preservation of implanted material.

Liu et al. [14] conducted a systematic review and meta-analysis of 36 comparative studies involving 3,681 patients with deep sternal wound infection after cardiovascular surgery. Compared with conventional wound management, NPWT was associated with lower mortality (risk ratio: 0.46, 95% confidence interval: 0.35-0.61) and reinfection risk (risk ratio: 0.43, 95% confidence interval: 0.25-0.74) [14]. However, the contributing studies predominantly involved post-sternotomy infections and were largely retrospective. Their findings therefore provide supportive but indirect evidence for an oncologic chest wall wound containing prosthetic material. In the present case, progression from exposed fixation material to a granulated wound suitable for flap coverage represents a favorable response.

Selection of vascularized soft tissue coverage

Definitive reconstruction required well-vascularized tissue capable of reaching the central anterior defect and covering the titanium bar and polypropylene mesh construct. Bakri et al. [15] describe the rectus abdominis as a type III muscle supplied by two dominant pedicles: the superior epigastric and deep inferior epigastric arteries. For chest wall reconstruction, it is commonly harvested as a superiorly based flap supplied by the superior epigastric artery. The flap is particularly useful for central anterior defects requiring substantial tissue volume, defects extending toward the xiphoid or epigastric region, and selected anterolateral defects when the latissimus dorsi is unavailable or insufficient [15]. In this patient, the inferior epigastric vessels were ligated and the rectus abdominis muscle was mobilized cephalad while retaining its superior attachment. Given the preceding bilateral internal thoracic artery ligation, collateral inflow through preserved intercostal and musculophrenic connections to the superior epigastric arterial network represents a plausible explanation for flap perfusion [7].

Banuelos et al. [16] reviewed 60 consecutive oncologic sternectomy reconstructions. The mean defect area was 148 ± 81 cm², and postoperative complications occurred in 14 (23%) patients. Although the pectoralis major was the principal flap used for sternal coverage, 19 (32%) patients required additional flaps, most commonly the rectus abdominis. Larger defects and resections involving the inferior sternum or overlying skin were significantly more likely to require additional tissue [16]. The reported dimensions of the present defect and prior involvement of the pectoral tissues support the anatomical rationale for using a rectus abdominis flap. Direct area comparison with the series by Banuelos et al. is inappropriate because the present defect was irregular and only its linear dimensions were documented. Furthermore, this case does not establish superiority over pectoralis major, latissimus dorsi, omental, or free tissue transfer alternatives. Graft integration of approximately 97% on postoperative day 5 and 98% on postoperative day 7, followed by clinical healing at approximately six months, represents a favorable early and medium-term reconstructive outcome.

Systemic therapy and oncologic follow-up

For histologically confirmed high-grade osteosarcoma, agents with established antitumor activity include doxorubicin, cisplatin, high-dose methotrexate, and ifosfamide [11]. Paclitaxel-carboplatin is not identified as conventional first-line therapy for localized high-grade osteosarcoma. However, because this patient had a poorly differentiated neoplasm without definitive osteogenic classification, the appropriateness of the administered regimen cannot be judged solely against osteosarcoma-specific recommendations.

The patient reported independence in his usual activities, no significant respiratory limitation during routine activity, and the ability to ride a bicycle comfortably. These observations indicate favorable patient-reported recovery but do not establish normalization of pulmonary function, chest wall mechanics, cardiac reserve, or exercise capacity. Aranda et al. [10] note that postoperative pulmonary function and quality of life have been evaluated in only a limited number of sternal reconstruction studies. Although chest CT at 8.5 months showed no reported residual or recurrent mass at the operative site, the limited duration and scope of surveillance preclude conclusions regarding durable oncologic control.

Extensive chest wall reconstruction remains technically demanding, whether it is performed for oncologic, infectious, or cosmetic reasons, and resections for cancer are best planned by multidisciplinary teams that include oncologists and thoracic and plastic surgeons [17]. In the present case, the thoracic surgery team performed the tumor resection and rigid stabilization, the plastic surgery team provided vascularized soft tissue coverage, and systemic therapy followed. This shows how such collaboration helps address oncologic clearance, chest wall stability, organ protection, infection control, and functional and cosmetic outcomes.

Limitations

This report is limited by its retrospective single-patient design, unresolved histologic classification, and documentation of only one systemic treatment cycle. Follow-up ended at approximately 8.5 months. Recommended radiotherapy could not be delivered because it was unavailable at the treating institution, and the available follow-up information was insufficient to determine recurrence, survival, or late implant-associated infection. Functional recovery was based on patient reports without objective pulmonary function, exercise capacity, pain, or quality of life assessments.

## Conclusions

Management of extensive sternal tumors requires coordinated oncologic resection, restoration of chest wall stability, and durable soft tissue coverage. This case demonstrates the feasibility of an individualized, staged multidisciplinary strategy incorporating infection control, rigid skeletal stabilization, and delayed vascularized soft tissue reconstruction when immediate definitive coverage is not feasible. However, the unresolved histologic classification, absence of objective functional assessment, and limited oncologic follow-up restrict conclusions regarding long-term tumor control and functional recovery. Further comparative studies are warranted to determine the optimal reconstructive approach for complex anterior chest wall defects.
